# Investigating the role of MMP9 in anti‐Abeta immunotherapy‐associated ARIA

**DOI:** 10.1002/alz70861_108871

**Published:** 2025-12-23

**Authors:** Kate E. Foley, Erica M. Weekman, Katelynn E. Krick, Jessica L Chalk, Donna M. Wilcock

**Affiliations:** ^1^ Indiana University School of Medicine, Stark Neurosciences Research Institute, Department of Neurology, Indianapolis, IN USA; ^2^ Indiana University School of Medicine, Stark Neurosciences Research Institute, Department of Anatomy, Cell Biology, and Physiology, Indianapolis, IN USA; ^3^ Indiana University School of Medicine Stark Neuroscience Research Institute, Indianapolis, IN USA

## Abstract

**Background:**

Amyloid related imaging abnormalities (ARIA) remain a major obstacle to the widespread use of anti‐amyloid immunotherapy. Data has indicated an association of neuroinflammation and subsequent MMP activation as being associated with anti‐amyloid immunotherapy. We therefore performed a co‐administration study of anti‐amyloid immunotherapy (3D6) with marimastat, an MMP inhibitor that targets MMP1, 2, 3, 7 and 9, with the highest affinity for MMP9.

**Method:**

We initiated treatment with both agents in 19 mo hAbetaSAA knockin mice. Monthly MRI imaging was performed and, upon euthanasia, we performed scRNAseq on the frontal cortex using a glial enrichment preparation, and histological analysis for microhemorrhages.

**Result:**

We found that anti‐amyloid immunotherapy‐mediated Prussian blue microhemorrhages were not significantly reduced by marimastat co‐administration. However, MRI detected microhemorrhages were reduced by marimastat treatment. In our scRNAseq dataset we found significant shifts in microglial state with anti‐amyloid immunotherapy not detected with the control IgG.

**Conclusion:**

MMP inhibition does not appear to impact small microhemorrhages detected histologically. However, the reduced microhemorrhages detected using MRI suggests that marimastat prevented the development of larger sized microhemorrhage events. Data analysis continues to gain further insights from this study.

